# A realist review of community engagement with health research

**DOI:** 10.12688/wellcomeopenres.15298.2

**Published:** 2019-08-02

**Authors:** Bipin Adhikari, Robin Vincent, Geoff Wong, Claire Duddy, Emma Richardson, James V. Lavery, Sassy Molyneux

**Affiliations:** 1 Centre for Tropical Medicine and Global Health, Nuffield Department of Medicine, University of Oxford, Oxford, OX1 3SY, UK; 2 Robin Vincent Learning and Evaluation Limited, Sheffield, UK; 3 Nuffield Department of Primary Health Care Services, University of Oxford, Oxford, OX2 6GG, UK; 4 Centre for Ethical, Social & Cultural Risk, St Michael's Hospital, Toronto, ON M5B 1W8, Canada; 5 Hubert Department of Global Health, Rollins School of Public Health, Emory University, Atlanta, Georgia, 30322, USA; 6 Center for Ethics, Emory University, Altanta, Georgia, 30322, USA; 7 Kenya Medical Research Institute (KEMRI) Wellcome Trust Research Programme, University of Oxford, Kilifi, 80108, Kenya

**Keywords:** realist review, community engagement, health research, low and middle income countries, malaria research

## Abstract

**Introduction**: Community engagement is increasingly recognized as a critical aspect of global health. Recent years have seen an expansion of community engagement activities linked to health research, but debates and inconsistencies remain about the aims of different types of engagement, mechanisms underpinning their implementation and impact, and influential contextual factors. Greater commitment to and consistency around community engagement by health research programs, implementers and funders requires a more coherent evidence base. This realist review is designed to improve our understanding of how and why community engagement contributes to intended and unintended outcomes (including research and ethical outcomes) in different contexts. Given the breadth and diversity of the literature on community engagement in health research, the review will initially focus on malaria research in low- and middle-income countries (LMICs) and draw on wider global health literature where needed.

**Methods and analysis**: Community engagement in practice is often a complex set of interventions. We will conduct a realist review – a theory driven approach to evidence synthesis – to provide explanations for how and why community engagement with health research produces the pattern of outcomes observed across different contexts of application. We will consolidate evidence from a range of documents, including qualitative, quantitative and mixed method studies. The review will follow several stages: devising an initial programme theory, searching evidence, selecting appropriate documents, extracting data, synthesizing and refining the programme theory, and reiteration of these steps as needed.

**Ethics and dissemination**: A formal ethics review is not required for this literature review.  Findings will be disseminated in a peer reviewed journal, through national and international conferences, and through a set of short briefings tailored for audiences with an interest in community engagement. Outputs and presentations will be informed by and feed into our network of community engagement experts.

**PROSPERO registration number:** CRD42019125687

## Introduction

### Background

Community engagement is increasingly recognized as a critical element of global health research, recommended by ethicists, funders and international ethics guidelines, such as the 2016 Council for International Organization of Medical Sciences (CIOMS) guidelines ^1– 3^. However, ‘community engagement’ remains a relatively ill-defined term with varied meanings and practices across the domains of health promotion, health related research, health programmes and international development ^4^. The diverse conceptual underpinnings of community engagement, the range of goals ascribed to it and wide variety of activities undertaken all complicate the evaluation of community engagement ^4^.

Community engagement has been defined as a process of collaborative work with groups of people affiliated by geographic proximity, interest or health issue, to address social and health challenges affecting those people ^5^. In practice in health-related research, community engagement encompasses a wide variety of activities and strategies, such as conducting meetings with community members and representatives, working with community advisory boards ^6, 7^ and involving members of the community in designing and implementing research activities ^4, 8^. Community Engagement also involves a wide range of different stakeholders in a dynamic set of social interactions with considerable relational complexity ^3, 9, 10^.

Our review aims to focus on CE in large research programmes in LMICs in order to understand how community engagement works in practice in such settings and assess claims made for contributions to research related and ethical outcomes. In addition to understanding ‘common current practice’ in community engagement in large health research programmes, we also want to understand potentially different dynamics developed through community engagement in other contexts in order to highlight alternative approaches and the distinctive mechanisms which may be brought into play in such settings.

### Goals and outcomes of community engagement

Scholars have identified a series of overarching goals of community engagement in health research ^2, 11^. A distinction is often made between the instrumental goals of improving the quality and relevance of research ^12^, including achieving recruitment and retention targets ^8, 11, 12^, and a range of ethical goals of community engagement, including: respecting individuals, communities and stakeholders ^3, 11, 13, 14^; building trust and social relationships ^9, 11, 15– 18^; determining appropriate benefits; minimizing risks, burdens and exploitation ^3, 4, 11, 19– 21^; supporting the consent process ^10– 12^; understanding vulnerabilities and researcher obligations ^3, 11, 23, 24^; and gaining permissions, approvals and building legitimacy ^3, 11, 14, 25, 26^.

Community engagement initiatives in health research often have more than one goal, however, and the distinction between instrumental and ethical goals in practice can be unclear ^10^. Further, studies that have attended to the relational dynamics of engagement, rather than formal ethical procedures ^15^, have made visible the ‘human infrastructure’ of research ^3^ and highlighted how a concern with ethical negotiation of relationships may be integral to achieving more instrumental research goals. Aspirational outcomes for community engagement with research do sometimes include community empowerment and community capacity building ^27– 29^. This is more common in participatory social development contexts, where there may also be a focus on some degree of co-production of the research itself ^30, 31^. However, in large health research programmes in LMICs, co-design of the research with communities from the outset and throughout all stages of the research process appears rare. The bulk of current literature on community engagement in global health focuses on the contribution of community engagement to research-related outcomes rather than the range of potential ethical outcomes, including the impact on stakeholder relationships.

Community engagement goals and activities are also potentially affected by the type of research and interventions in which initiatives are embedded and the opportunities and constraints for engagement that they entail ^4, 8, 32^. For instance, in mass drug administration studies and vaccine studies, where study success depends upon a high population coverage, community engagement may be emphasized to achieve study related aims rather than other goals ^8, 33, 34^. In hospital based clinical trials, community engagement may focus on engaging with patients at the hospital rather than broader communities. There are guidelines for good participatory practice in clinical trials aimed at maximising the meaningful involvement of individuals and communities ^28^, but some have nevertheless argued that the nature of clinical trials can circumscribe the scope for community involvement compared to other forms of social and health research ^35^.

### Community engagement mechanisms

**Figure 1.**
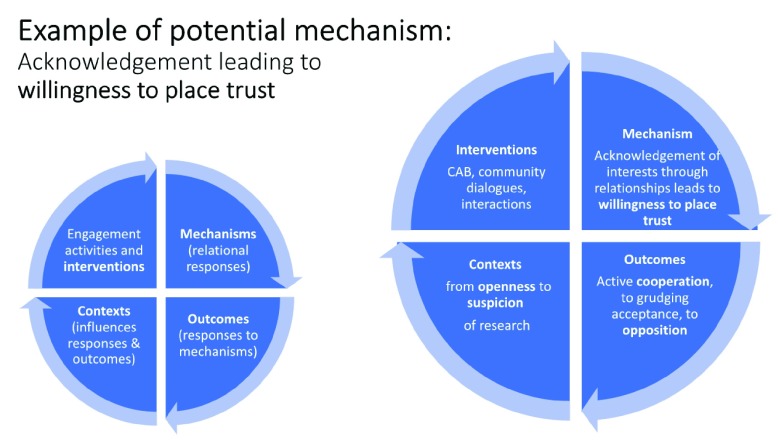
Example mechanism.

From a realist perspective, diverse community engagement activities may rely on a more limited number of relational mechanisms. Potential mechanisms hinted at in the literature include: respectful interactions facilitate trust ^9, 16^; listening and acting to show understanding and express recognition, leads to a sense of people being heard ^11, 16^; and responsiveness of research processes increases motivation to participate ^14, 16, 36– 38^. A range of different engagement activities may contribute to a mechanism coming into play, and differences in context will affect how these mechanisms work in particular settings, producing a pattern of outcomes ^39, 40^. Figure 1 provides a simplified example.

At the same time, several different mechanisms may work in combination, some of which may contribute to intermediate outcomes that, in turn, become an important new context within which other mechanisms may operate. Drawing on some of the potential mechanisms highlighted in the above paragraph, we can see how they may combine and interact with each other (recognising that whether or not they do in practice is an empirical question for the review to consider). For example, building of relationships between researchers and community members may help to establish trust and the credibility of research. At the same time, where community members can provide feedback and voice their concerns about research through meetings and discussions, and have these acknowledged and responded to, they may feel valued and respected and this may also contribute to the establishment of trust. Established trust may be an important precursor to a collaborative partnership between researchers, community members and other stakeholders, which may in turn enhance community members’ sense of ‘being a part of’ the research and may create a sense of responsibility and ownership in the research. In this way, engagement is likely to involve a number of different mechanisms in combination and their sequencing may prove to be important.

### Influence of context

While some of the above mechanisms may be common across a wide range of types of health research, whether or not they come into play may be affected by a particular local social and cultural context ^8, 40^. For instance, the impact of community engagement on targeted malaria elimination studies that aims at wide community mobilisation may be enhanced in a cohesive community ^14^ compared to a politically fragmented community ^38, 40^. In recent years, the details of social and cultural context have been increasingly reported in accounts of community engagement around health research ^14, 36, 37, 41, 42^. For example, the geo-political context of Thai-Myanmar border, livelihoods based on subsistence farming, the influence of traditional healing practices and remote and limited health service provision have been highlighted as affecting engagement outcomes ^38^. A recent systematic review of the last century’s literature on community engagement and population coverage in mass antimalarial administration identified a range of contextual factors ^8^, including: how community engagement and population coverage could be affected by the social cohesion of the communities ^33^; the influence of social hierarchies ^14^ and political factions ^34, 36, 38^; cultural beliefs around blood tests (including rumours such as blood stealing) ^14, 16^; the influence of traditional healing practices ^43^; and, more broadly, perceptions around the concept of research ^37^, its rationale and the impacts ^42, 44^.

To date, however, there is a lack of analysis and explanation of how the factors of local social and cultural context have affected particular outcomes. Similarly, as noted above, a number of potential mechanisms have been hinted at in the literature, but these are often left implicit without any systematic analysis, including how they work in combination and are influenced by context.

### Addressing diversity

Realist review and synthesis provides a framework to look at configurations of context, mechanism and outcome and to draw out regularities and patterns that endure across context ^45, 46^. Some of the team involved in the current review previously attempted to conduct a conventional systematic literature review, but faced great challenges due to the extraordinary complexity and diversity of the community engagement literature ^47^. They recommended that a realist review may be better suited to address such complexity ^47– 50^. Using a realist approach, the current review will focus initially on malaria research to explore: the intended (and unintended) outcomes of community engagement; mechanisms by which community engagement strategies are believed to generate the outcomes; and important contexts that affect various mechanisms and outcomes. The ultimate aim of this review is to develop transferrable learning about community engagement in health research and programmatic interventions.

## Methods

### Glossary of terms

Realist review utilizes specific realist concepts with particular meanings, which are briefly described here ^51^.

**Context:** It refers to the backdrop of programs and research. For example, context can include cultural norms and history of the community in which a program is implemented, scope and the extent of existing social networks or the infrastructure in which the programme is built. They can be trust-building processes, geographic location effects, funding sources, opportunities or constraints. Context can thus be broadly understood as any condition that trigger and/or modifies the behaviour of a mechanism.

**Mechanism:** There are many definitions of mechanism. What they have in common is that mechanisms generate outcomes. Mechanisms are the agents of change. They describe how the resources embedded in a programme influence the reasoning and ultimately action of programme ‘subjects’. Mechanisms are underlying entities, processes, or structures which operate in specific contexts to generate outcomes of interest. Mechanisms are usually hidden and are sensitive to variations in context and generate outcomes.

**Outcome:** The intended and unintended results of interventions or programmes. Complex programmes may involve intermediate outcomes that provide important pre-conditions for other outcomes to be realised.

**Programme theory:** A programme theory is “an abstracted description and/or diagram that lays out what a program (or family of programs or intervention) comprises and how it is expected to work”. It is usually made up of a set of interlinked propositions explaining the causal relationships involved in how a programme works in practice

**Context-mechanism-outcome configurations (CMOc):** A CMO configuration explains the casual relationship between a particular aspect of context, whether or not a mechanism of interest is triggered by it, and the outcomes produced. A programme theory usually combines a range of different CMOcs, which may overlap or be nested to explain the observed patterns in phenomena. Configuring CMOs is a fundamental process in realist review to generate or refine the theory that becomes the final product of the review.

### Review aim

This review aims to improve our understanding of the ways in which (i.e. how, why and in what contexts) community engagement interventions contribute to (or do not contribute to) reported outcomes (both explicitly aimed for and unintended), including the range of research related and ethical outcomes highlighted above under the section ‘Goals and outcomes’, and the roles of context and mechanisms in each case. Given that outcomes are not always clearly articulated in reports of community engagement, the review will also consider revealed outcomes, again attempting to understand the influence of particular mechanisms and context.

Our review will focus initially on large malaria research programmes to understand ‘common current practice’ in community engagement in settings of global health research. In addition, we may draw on wider literature to better understand some of the mechanisms underpinning community engagement outcomes such as empowerment and community capacity building, where adequate data cannot be found in the literature on large research programmes.

The review will focus initially on malaria research because it is a major strand of global health research, it is an area where members of the review team have experience and knowledge as well as access to practitioners and expertise, and it provides a pragmatic way of focusing the review to make it manageable.

### Review objective

To conduct a realist review to understand the ways in which community engagement contributes to intended and unintended outcomes, and through which key mechanisms. This will be done with (A) engagement with a diverse range of literature, (B) the development of a programme theory and (C) feedback and advice from stakeholders experienced in the field.

### Review research question

Within the existing and available literature pertinent to malaria research, what are the causal explanations for the ways in which community engagement contributes to intended and unintended outcomes?

Sub-questions:

1. What are the intended and unintended outcomes of community engagement strategies?
2. What are the key mechanisms by which community engagement strategies result in their intended and unintended outcomes?
3. What are the important contextual influences on the ways in which different mechanisms produce intended and unintended outcomes?

### Study design

Realist review is increasingly recognized as an effective process for consolidating evidence and learning from complex social programming, particularly in public health and community development ^48– 50^. The proposed review design is based on the realist review approach ^46^, which aims to explore how community engagement produce outcomes in specific contexts through the operation of a number of key mechanisms. This review will initially focus on the community engagement embedded in malaria research in low and middle income countries. The initial search commenced on 21 ^st^ February and screening from 12 ^th^ March, 2019. We anticipate the review will be completed by the 30 ^th^ June, 2020.

Over the years, realist reviews have explored the configurations of context, mechanisms and outcomes for complex issues, such as antimicrobial prescription by doctors ^52^, access to primary care for socio-economically disadvantaged elderly populations in rural areas ^53^, and the process of appraising the performance of doctors ^54^. Because community engagement activities are complex interventions that work through a variety of different mechanisms to produce different outcomes across different contexts, realist review holds the promise of bringing greater clarity and understanding to the variation of engagement in practice.

A realist review eschews the traditional hierarchy of evidence and incorporates a wide variety of data, including those derived from qualitative, quantitative, mixed methods studies, as well as grey literature. The review will include all the pertinent documents published in English language only. In contrast to a more conventional review, in which the data are extracted and aggregated across a selection of studies deemed to be of sufficient quality, in realist review, diverse data are drawn upon depending on their potential value to contribute to refinement of programme theory. Within realist reviews assessments are not made for risk of bias of individual studies or meta-bias across studies ^55^. Findings derived from this realist review are expected to be transferable because they will focus on commonly occurring mechanisms through which community engagement produce both intended and unintended outcomes. This will enable us to produce recommendations likely to be useful across domains beyond malaria within global health research. For the sake of clarity we have set out the realist review as six distinct steps. However, as is common in many realist reviews, our expectation is that we may need to make changes to what we have initially planned and set out in our protocol in order to answer our review questions ^56^. Finally, we acknowledge that realist reviews incorporate iterative cycles within the steps outline below and of engagement with the literature and with stakeholders with relevant knowledge.

### Stakeholder engagement

**Figure 2.**
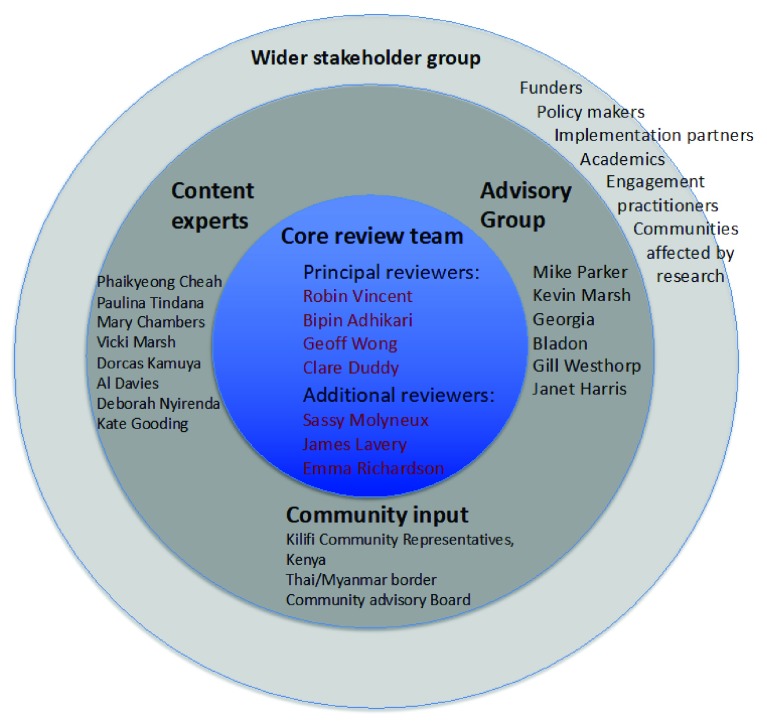
Review team and stakeholder input.

We aim to involve a wide range of stakeholders with expertise and experience of community engagement. At key stages in the review these stakeholders will provide input and feedback on relevant papers, our literature search strategies, and evolving learning. Figure 2 shows the structure of inputs into the review. We have established a ‘content expert advisor’ group comprised of experts from low- and middle-income countries (LMICs) who are community engagement practitioners and scholars. This group has been involved in the generation of the initial candidate programme theories for the review, and will provide input at key moments throughout the process. Our advisory group draws on expertise in bioethics, malaria, realist review and includes health research funders. In addition, we aim to solicit input from community members involved in community engagement processes, including in Kenya and on the Thai/Myanmar border. In both of these contexts, core team members and content experts are able to support this process. The website for the review is a programme hub within the MESH community engagement website [ https://mesh.tghn.org], which brings together a large LMIC based network including over 1000 practitioners of community engagement. We aim to use this platform to draw in and consult CE practitioners. An evolving group of wider stakeholders, including academics, policy-makers, funders, research programme managers, implementers and engagement practitioners, will also provide input. The wider stakeholder group will be identified using a snowball approach through the authors’ professional networks, engagement at conferences, and through more systematic outreach based on identifying stakeholders from different disciplines, identified in literature and relevant programming initiatives.

### Step 1: locating existing theories

As a first step in realist review, an initial scoping search is conducted to identify theories that begin to explain and develop our understanding of community engagement. This stage is crucial to visualize the underpinning assumptions about why certain components and processes of community engagement are required to achieve one or more desired outcomes ^45^.

At the initial stage, these theories are explored in two main ways: 1) drawing on exploratory searches of relevant literature in repeated cycles; and 2) consulting with key content experts who have practical experience of implementing community engagement.

**Figure 3.**
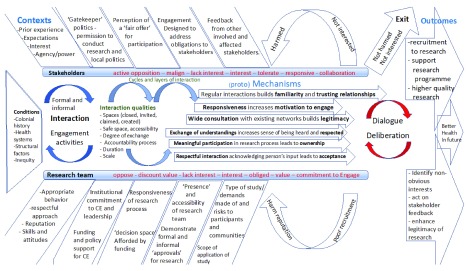
Initial programme theory visualisation.

The first stage of our initial scoping of key literature to identify elements of programme theory involved reviewing key literature in the field of community engagement recommended by our content experts. A summary of commonly recognised outcomes of community engagement, potential mechanisms and important elements of context was then discussed among the core team and our context experts to produce elements of an initial programme theory and related visualisation. This was subsequently refined through further discussion among the team. Figure 3 is the consolidated visualisation of elements of an overall programme theory that was then used to help focus our search strategy. It should be noted that at this stage the diagram is more a summary collection of still (heterogenous) elements, not strictly configured or consistent (where the labelling of context, proto-mechanisms and outcomes is only provisional), and that some of the terms are indicative at this stage rather than strictly defined (since their meaning in practice will depend on how they are evidenced in the literature). In addition, the visualisation includes an initial sense of the distinction between more background ‘conditions’ and the more active elements of ‘context’ which are expected to have a more salient influence on the mechanisms of interest. Making putative contexts, mechanisms and outcomes as explicit as possible is an important first step to help focus our ongoing systematic literature searches. This stage was not exhaustive but helped to capture key aspects of engagement and to help focus more systematic literature searches in stage 2.

**Figure 4.**
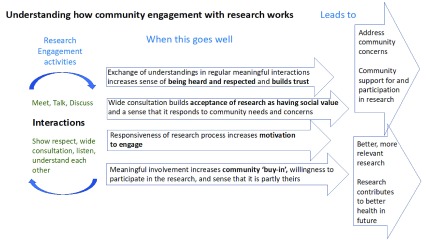
Simplified programme theory for consultations.

This initial set of elements gathered in the programme theory visualisation ( Figure 3) will be further simplified to produce a version that can be used in community consultations in Kenya and the Thai Myanmar border. Figure 4 shows an early iteration of a simplified version of the diagram, notable for the way it focuses on our initial sense of potential mechanisms and omits aspects of context. A further refined version of this visualization may be used in addition to some open-ended questions as part of consultation with community representatives, to both solicit community perspectives, but also ‘test’ this initial sense of what may be important in community engagement. Depending on how plans for the community consultations develop, it may or may not be that the use of such a visualisation is considered to be helpful. We include it here to provide a simplified illustration of some of the expected key relational mechanisms at the outset of the review.

Further development of programme theory will be iterative and incorporate insights from discussions within the project team and advisors, and findings emerging from review of the literature. Regular meetings will be conducted with the aim of challenging, sense-making and synthesizing a range of different elements into particular configurations of context, mechanism and outcome, and integrating these into an overall programme theory.

### Step 2: searching for evidence

We anticipate that searching for evidence will involve literature searches in up to three broad categories. The first search will identify literature that describes or discusses the common current practice of community engagement in large research programmes, focusing on clinical trials (for example, mass antimalarial administration and malaria vaccine trials) in LMICs as defined by World Bank criteria. This first search will include a supplementary search based on citation chaining of key international ethical guidance and policy documents on community engagement with clinical trials. If more relevant data are needed then our second search will involve identification of literature that describes community engagement practices in other research paradigms and across disciplines. In addition, the searching will be flexible and if needed will incorporate relevant studies published up to the completion of the review. This search will initially still focus on community engagement in malaria, but we will loosen this requirement if additional relevant data are still needed for programme theory development and testing. Finally, if needed, the third search will involve identification of literature that discusses failure, challenges and problems in community engagement. In addition to searches of electronic bibliographic databases, techniques including forward and backward citation chaining, and methods to identify ‘kinship’ or ‘sibling’ studies of relevant documents will also be employed after the screening to help identify the relevant materials ^51^.

The goal of such a search strategy is to identify adequate literature that can further inform the development of a more detailed programme theory. The process of designing, piloting and conducting the formal searches will be conducted with the support of an information specialist (CD). Modifications and adaption of search strategies following the pilot will be documented and implemented across source types.

The following electronic bibliographic databases will be searched: MEDLINE, Embase, Global Health, CINAHL, The Cochrane Library, the Web of Science Citation Indexes (Core Collection), Scopus, the Global Index Medicus, IBSS and ASSIA. Additional databases identified by the information specialist may be added later. Informed by the initial programme theory developed in step 1, each search strategy will be built around two main concepts: malaria and community engagement. Additional terms will be added to identify literature in the three categories described above. A comprehensive set of free text and subject heading terms will be used to identify the relevant documents. Search terms will be chosen based on key documents identified by the project team and wider content expert groups, discussion in these groups, and the initial programme theory. The searches will not be limited by the date and will be limited to English language. Searching is underway, and the search strategies employed for the first of the planned searches are presented as *Extended data* ^58^.

All screening will be undertaken by BA. RV will screen a random sample of 10% of records to support discussion and refinement of inclusion criteria. Disagreements and consistency between these two reviewers will be discussed amongst themselves first and with the project team members if and when necessary.

Initial screening will be conducted based on the title, abstract and keywords. We will use following inclusion criteria to determine if a document is likely to contain the relevant data:

➢ Community engagement in malaria research. By community engagement, we are predominantly referring to the range of strategies undertaken alongside research, for example meetings and discussions with the stakeholders, and training and devolvement of responsibilities to community volunteers.
➢ Document type: all study designs and documents that may contain relevant data.
➢ Types of (participants) studies: documents that include research focused on community engagement or community engagement embedded in any malaria related research.
➢ Types of intervention: community engagement conducted alongside research to promote research and ethical outcomes or relevant case studies of community engagement in long established research institutions.
➢ Outcome measures: both intended and unintended outcomes of community engagement will be explored, for example: (1) research outcomes - recruitment, support for research programme and higher quality research; and (2) ethical outcomes - identification of non-obvious interests, acting on stakeholders’ feedback, and enhanced legitimacy of research.

During the screening process, documents will be excluded based on their content using following criteria:

➢ Research documents which have only briefly mentioned community engagement but with no further details on how the community engagement was conducted, what it entailed or related outcomes.

### Additional searching

As the aim of the realist review is to include a broad range of documents to further inform the development of the programme theory, where needed we will look across disciplines, outside of malaria and in different research paradigms, particularly in relation to the exploration of mechanisms for community engagement to produce both intended and unintended outcomes of interest. For example, we may undertake additional searches as the programme theory develops for a number of reasons: to fill in evidence gaps; develop understanding of potential mechanisms; and borrow analogies or theories from other relevant disciplines.

### Step 3: document selection

Documents included from screening of titles and abstracts will be considered for selection into the review. The full text of documents initially screened into the review will be further sorted and selected for inclusion primarily based on two criteria: 1) relevance in terms of how and to what extent they can contribute to the programme theory development and refinement; and 2) rigour, which refers to the credibility and trustworthiness of methods used to generate the data ^45^. Documents pertinent to community engagement in clinical trials of malaria or programmes will be initially prioritized for inclusion and analysis. Other health research with relevant information on community engagement will also be subsequently incorporated based on its’ potential to strengthen our understanding of community engagement processes. These papers will be categorized as having potentially major or minor contributions to answering the research questions.

Major contributions include:

➢ Documents which contribute to answering the research questions and conducted in the field of clinical trials related to malaria in LMICs
➢ Documents which contribute to answering the research questions but are not focused on clinical trials; for example, descriptive account of community engagement strategy in a research institution.
➢ Documents which do not focus on malaria, but health research, for example HIV, tuberculosis or Ebola research, which can significantly inform our review in terms of understanding the processes and mechanisms.

Minor contributions include:

➢ Documents which report community engagement in high income countries, non-clinical trial contexts, for example development science, but where the mechanism could plausibly operate in the circumstances of LMICs.

This process, together with the developing discussions around the literature, will enable reviewers to focus on data extraction and analysis of papers that provide a conceptually rich contribution, while still including documents that are less conceptually rich.

### Step 4: data extraction

Following the methods outlined in a previous realist review ^59^, data extraction will take place in two stages. At first, the selected documents including their characteristic details will be extracted into a table. This will provide a descriptive account of the documents included. In the second stage, all the selected documents will be analysed using NVivo. Extraction of data will be undertaken first by BA and will be independently reviewed by RV for consistency and refinement of the codes. Discussions will be held with the core project team members for consistency of codes, interpretation and the (interim) findings. Discussions will be held with the wider project team when there are disagreements within the project team which cannot be resolved. Any discussions and their outcomes among core team members and the wider project team members will be recorded.

### Step 5: data synthesis

The main aim of the data synthesis in realist review is to develop and then confirm, refute or refine parts of the programme theory. The initial programme theory will be further developed by drawing on the data found within included documents. Analysis of the data will entail using a combination of various methods of reasoning that includes a deductive approach, in which the codes used to code data are based on the initial programme theory, an inductive approach in which codes will emerge from the documents reviewed and a retroductive approach, where inferences are made based on interpretations of the data contained within included documents of underpining mechanisms.

Analysis will following the process set out by Papoutsi *et al*. ^60^. Primary reviewers BA and RV will develop initial CMOCs and these initial CMOCs together with the emerging CMOCs will be discussed amongst the core team for validation and refinement.

The review will follow the standard Realist and Meta-Review Evidence Synthesis: Evolving Standards (RAMESES) guidelines on quality and reporting ^61^.

### Step 6: refine programme theory

The final step in realist review is the refinement and validation of the programme theory ^46^. To ensure that the final programme theory makes pragmatic sense, experts and practitioners of community engagement will be consulted to inform final refinement of the programme theory ^62, 63^. Incorporating the inputs of community members and community engagement practitioners (field staff) will help us to make it practical and realistic. We also aim to hold an international validation workshop approximately 15 months into the review, in order to validate the findings but also to gather input to help develop tailored versions of the findings for a number of different audiences, including funders and policy-makers and global health research managers and engagement practitioners ^46^.

Towards the end of this process of refinement, the review team will revisit parts of the review that require re-scrutinizing. This process will be continued until no new information is provided by the evidence or stakeholder involvement, essentially reaching theoretical saturation ^46^.

### Strengths and limitations

- To our knowledge, this is the first realist review aiming to synthesize evidence and produce conceptualizations on community engagement with health research in LMICs.
- A realist review should enable us to understand the complexity of community engagement and its outcomes in diverse contexts. The programme theories developed through the process should be relevant across contexts.
- Stakeholder engagement during programme theory development will ensure a range of perspectives inform the review and support the relevance and uptake of the findings.
- The breadth and diversity of the community engagement literature remain a challenge. Our review will use community engagement with malaria research as a pathfinder topic and will draw on wider literature on community engagement where necessary.

### Ethics and dissemination

Ensuring that the outputs from this review are useful to the community engagement practice in health research is a key priority for us. Aligning with this value, we will produce relevant and appropriate outputs that target a range of audiences, in conjunction with stakeholder consultation:

1. Academic forums: we aim to publish in a high-impact peer-reviewed journal including sharing our work in relevant academic conferences where inter-disciplinary scientists attend.
2. Plain English summaries and briefing documents: apart from the academic outputs, we will produce documents which will be simplified for a range of particular audiences, including for a non-expert audience, with an aim of maximum uptake and dissemination of our evidence.

### Study status

The study has piloted the preliminary search strategy and the number of studies resulting from this initial search are undergoing screening and has not been completed yet.

## Data availability

### Underlying data

All data underlying the results are available as part of the article and no additional source data are required.

### Extended data

Harvard Dataverse: Replication data for a realist review of community engagement with health research. https://doi.org/10.7910/DVN/DZ4B2Z ^58^

This project contains the following underlying data:

- Appendix 1.docx (database search strategies for preliminary searches)

Data are available under the terms of the Creative Commons Zero “No rights reserved” data waiver (CC0 1.0 Public domain dedication).
