# Supplementary material for: A realist review of community engagement with health research
Source: Wellcome Open Res. 2019 Aug 2;4:87. Originally published 2019 May 31. [Version 2] doi: 10.12688/wellcomeopenres.15298.2 (PMC6611131; doi:10.12688/wellcomeopenres.15298.2)
Supplement: Peer review history

# Peer review: Reviewer response for version 2

**DOI:** 10.21956/wellcomeopenres.16812.r36125

**Published:** 2020-01-09

**Authors:** Maxine Whittaker

**Affiliations:** 1 Division of Tropical Health and Medicine, James Cook University, Townsville, Qld, Australia

I confirm that I have read this submission and believe that I have an appropriate level of expertise to confirm that it is of an acceptable scientific standard.

# Peer review: Reviewer response for version 2

**DOI:** 10.21956/wellcomeopenres.16812.r36124

**Published:** 2019-08-14

**Authors:** Suzanne Day

**Affiliations:** 1 Institute for Global Health and Infectious Diseases, University of North Carolina at Chapel Hill, Chapel Hill, NC, USA

I have read the authors' responses to each of my concerns, and I believe they have given them thorough consideration and addressed them sufficiently in the revised version of the submission.

I confirm that I have read this submission and believe that I have an appropriate level of expertise to confirm that it is of an acceptable scientific standard.

# Peer review: Reviewer response for version 2

**DOI:** 10.21956/wellcomeopenres.16812.r36126

**Published:** 2019-08-09

**Authors:** Tracey Chantler

**Affiliations:** 1 London School of Hygiene and Tropical Medicine, London, UK

I am happy with the revisions that have been made.

I confirm that I have read this submission and believe that I have an appropriate level of expertise to confirm that it is of an acceptable scientific standard.

# Peer review: Reviewer response for version 1

**DOI:** 10.21956/wellcomeopenres.16697.r35705

**Published:** 2019-07-24

**Authors:** Tracey Chantler

**Affiliations:** 1 London School of Hygiene and Tropical Medicine, London, UK

This is a very interesting study protocol and the research that ensues will make a positive contribution to the field. 

 I have several comments however some of which relate more to the study design, hence may be difficult to change but aspects could be used to improve the paper and possibly refined the methodology. 

1. I think that the rationale for using a realist approach needs to be summarized earlier in the paper. Some detail in provided in the methods section however terms relevant to realist evaluation are used earlier in the paper, hence need to be defined at an earlier stage. It would be good to have a text box providing information about this in addition to the Figure 1 example of a mechanism. With reference to this, it can be difficult in practice to distinguish between an intervention/activity and a mechanism. This highlights the importance of providing a full explanation of realist evaluation theory at an early stage of this paper. 
2. In your aims and objectives you state that you will develop a programme theory. This needs to be explained. Normally each intervention/CE strategy would have there own programme theory that accounts for the context, the problem, what change is be activated and how this will be measured in terms of outcomes. If I understand correctly you are trying to develop a theory for CE strategies and maybe seeking to identify what are the main mechanism that support 'effective' CE in different contexts. Again mechanisms may vary significantly across contexts, cultures and also be affected by the institutional cultural of the organisations involved. 
3. Could you justify more clearly why you are focusing on malaria research. This may have just been a pragmatic decision but please clarify. 
4. The stakeholders involved are mainly based at international health research centers - I wonder whether this may result in this work focusing more on CE in these settings rather than more broadly. It is great that you have representation across different institutions, continents and countries, but some areas of the world are missing. This could be due to you focus on malaria research. Thinking about Haiti in the Caribbean and parts of South America.  

I confirm that I have read this submission and believe that I have an appropriate level of expertise to confirm that it is of an acceptable scientific standard, however I have significant reservations, as outlined above.

# Response

**Published:** 2019-07-26

**Authors:** Bipin Adhikari

**Affiliations:** University of Oxford, UK

Dear Tracey,

 We are grateful to your suggestions and comments. Your suggestions were very helpful and we believe that these suggestions have improved our manuscript significantly.

 We have added our responses below with relevant corresponding changes in the manuscript.

 We look forward to your kind consideration.

 Sincerely yours,

 On behalf of co-authors,

 Bipin Adhikari

**This is a very interesting study protocol and the research that ensues will make a positive contribution to the field.**

****

**I have several comments however some of which relate more to the study design, hence may be difficult to change but aspects could be used to improve the paper and possibly refined the methodology. **

1. **I think that the rationale for using a realist approach needs to be summarized earlier in the paper. Some detail in provided in the methods section however terms relevant to realist evaluation are used earlier in the paper, hence need to be defined at an earlier stage. It would be good to have a text box providing information about this in addition to the Figure 1 example of a mechanism. With reference to this, it can be difficult in practice to distinguish between an intervention/activity and a mechanism. This highlights the importance of providing a full explanation of realist evaluation theory at an early stage of this paper.** 

 

 AUTHORS: Thanks for the comments. We have added an explanation of what a realist programme theory is and tried to explain more clearly the initial and provisional nature of our programme theory visualisation. The added glossary of terms also aims to bring additional clarity to key realist terms.

  

1. **In your aims and objectives you state that you will develop a programme theory. This needs to be explained. Normally each intervention/CE strategy would have there own programme theory that accounts for the context, the problem, what change is be activated and how this will be measured in terms of outcomes. If I understand correctly you are trying to develop a theory for CE strategies and maybe seeking to identify what are the main mechanism that support 'effective' CE in different contexts. Again mechanisms may vary significantly across contexts, cultures and also be affected by the institutional cultural of the organisations involved. **
**  **

AUTHORS: Thanks for the comment. Initial scoping of the literature suggested that there may be a smaller set of relational dynamics that underpin the much wider variety of engagement interventions and activities. We do expect the mechanisms to have some relevance across context but also, as is key to realist reviews, aim to ascertain where particular contexts make a difference to how they do or do not come into play and in what combination

  

1. **Could you justify more clearly why you are focusing on malaria research. This may have just been a pragmatic decision but please clarify. **

 

 AUTHORS: Malaria is both an important are of health research in its own right, and a key area of practical experience for members of the team. But as the reviewer indicates, it is also a way, pragmatically, to focus the review initially in order to make it manageable and achievable.

  

1. **The stakeholders involved are mainly based at international health research centers - I wonder whether this may result in this work focusing more on CE in these settings rather than more broadly. It is great that you have representation across different institutions, continents and countries, but some areas of the world are missing. This could be due to you focus on malaria research. Thinking about Haiti in the Caribbean and parts of South America.  **

 

 AUTHORS: Thanks for the comment. Indeed, the initial focus of the work is on large programmes of international health research given the bulk of engagement work undertaken in this type of setting. We hope this will allow an understanding and assessment of some of the claims being made for engagement in what is a predominant context for engagement in global health research. As noted in responses to other reviewers, there will be both benefits and limits to this particular focus which we will aim to clearly document. We also aim in our Search B to look beyond this particular setting to consider how engagement, and the mechanisms and contexts involved may differ. The point about stakeholder relationships is an important one, and we will seek to systematically seek input from different geographical regions as we develop the stakeholder network.

# Peer review: Reviewer response for version 1

**DOI:** 10.21956/wellcomeopenres.16697.r35657

**Published:** 2019-07-02

**Authors:** Sanjeev Sridharan

**Affiliations:** 1 Bill and Melinda Gates Foundation, Delhi, New Delhi, India

I am enthused about the prospects of conducting the realist synthesis on community engagement. I think the field can benefit from such a study. The team has the right mix of competencies to successfully complete a realist synthesis of a complex area such as community engagement.

 Despite my enthusiasm for such a realist synthesis, I am not fully convinced that the approach as proposed will generate useful knowledge to help plan more meaningful community engagement for global health research. Key concerns that I have include the following points:

1. **Defining ‘outcomes’ from a community lens: **Perhaps my primary concern is that the paper provides very little focus on defining what “outcomes” actually mean. There is little to no discussion on what outcomes would be measured to understand benefits to the community. This is a significant oversight. The promise of a realist perspective in this study is its potential to generate knowledge of the context and mechanisms that might be associated with community engagement. Because the proposed protocol makes little effort to define outcomes from the perspective of the community, I am a little concerned that this study might not generate useful, actionable information.
2. **Understanding of the community engagement process**: I found very limited discussion on what is community engagement. The process of community engagement is not discussed. I would have ideally preferred a little greater clarity on the *dynamic* nature of community engagement. As it presently reads, the construct of community engagement is under-theorized.
3. **Towards deeper understanding of the multiple shades of community engagement**: Given the formidable complexities that might underlie community engagement, I am sensitive to the challenges of understanding the CMO configurations underlying community engagement. I would recommend a more thoughtful discussion on why community engagement around research in malaria might be a little different than community engagement for other goals (for example, community engagement for gender empowerment). I found the discussion around the concept of community engagement quite mechanical.
4. **A more explicit focus on LMIC contexts**: Given that the research is being doing in LMIC settings, I did not find a discussion about the challenges of conducting research in LMIC setting. I found myself wondering which specific contexts in such settings will be explored. In what ways are the mechanisms associated with community engagement different in LMIC contexts?
5. **The need to more explicitly focus on values**: Underlying much community engagement is a set of values. Community engagement can be quite exploitative or can be very respectful recognizing that the community is the seat of the decisions. The present version of the protocol takes a very instrumental approach to community engagement. I was somewhat surprised by an absence of any discussion around values in a realist synthesis protocol on community engagement.
6. **Taking unintended outcomes seriously**: There is a claim that the review will explore unintended outcomes, but there is very little discussion on how the authors plan to explore this. Most evaluations that might form the basis of such a synthesis do not often report on unintended outcomes; in this light it’s not clear how the authors intend to report on a synthesis of unintended outcomes.
7. **The need for prioritization**: Given the complexity of community engagement, wide varieties of CMO configurations might be possible. In the absence of clear prioritization, the complexity of having many CMO configurations with potentially limited evidence (across the multiple configurations of contexts and mechanisms) needs to be more explicitly discussed.
8. **Interrogating the theory of change**: Much of the theorizing is from a researcher’s perspective. The review presently does not have a strong plan to incorporate a community perspective to challenge, interrogate and refine the program theory. For example, I would have liked the authors to more clearly discuss how the community group members within the Wider Stakeholders Group can be utilized to refine the theory of change. 

**Comments on Rationale**

 While the rationale for the study is well described, it’s unclear in the absence of information on the outcomes how the proposed realist synthesis plans to achieve the objectives of the study. As pointed out earlier, the study also suffers from taking very much of a researcher’s perspective on potential benefits of research without sufficiently incorporating community members in either clarifying what community engagement means or in refining the theory of change.

**Appropriateness of Study**

 While there is much to be enthusiastic about the application of realist synthesis in understanding community engagement, many aspects of the proposed approach need to be further refined. The lack of a clear prioritization of CMO configurations, under-theorization of the dynamic nature of community engagement, and an absence of the discussion of the LMIC context that might be appropriate reduce my confidence in the proposed approach’s ability to refine and develop a useful program theory for community engagement. I would recommend focusing on some of these points.

**Details of the methods**

 As it presently reads, the details of the realist synthesis follow a general cookie-cutter approach and the complexity of conducting a realist synthesis for a complex area like community engagement especially in the domain of malaria research is not sufficiently considered. I also would argue that the Figure 3 as presented is not sufficiently clear as the starting point for developing an initial program theory.

I confirm that I have read this submission and believe that I have an appropriate level of expertise to confirm that it is of an acceptable scientific standard, however I have significant reservations, as outlined above.

# Response

**Published:** 2019-07-26

**Authors:** Bipin Adhikari

**Affiliations:** University of Oxford, UK

Dear Sanjeev,

 We are grateful to your suggestions and comments. Your suggestions were very helpful and we believe that these suggestions have improved our manuscript significantly.

 We have added our responses below with relevant corresponding changes in the manuscript.

 We look forward to your kind consideration.

 Sincerely yours,

 On behalf of co-authors,

 Bipin Adhikari

 I am enthused about the prospects of conducting the realist synthesis on community engagement. I think the field can benefit from such a study. The team has the right mix of competencies to successfully complete a realist synthesis of a complex area such as community engagement.

 Despite my enthusiasm for such a realist synthesis, I am not fully convinced that the approach as proposed will generate useful knowledge to help plan more meaningful community engagement for global health research. Key concerns that I have include the following points:

1. **Defining ‘outcomes’ from a community lens:** **Perhaps my primary concern is that the paper provides very little focus on defining what “outcomes” actually mean. There is little to no discussion on what outcomes would be measured to understand benefits to the community. This is a significant oversight. The promise of a realist perspective in this study is its potential to generate knowledge of the context and mechanisms that might be associated with community engagement. Because the proposed protocol makes little effort to define outcomes from the perspective of the community, I am a little concerned that this study might not generate useful, actionable information.**

 

 AUTHORS: Thank you for highlighting the importance of outcomes from the perspective of communities being engaged. As noted in our response to reviewer 3 we may have not made clear enough how the search strategy in the review aims to access and understand such potential outcomes. While our initial searches (search A) focus on large health research trials, and these may pick up community input into research protocols, informed consent processes and benefits of research participations, community perceptions of researchers, there may still be little emphasis in this setting, on more emancipatory outcomes for the community. Our search B aims to help elucidate mechanisms around community empowerment by looking at examples beyond common current practice in large clinical trials. However, this needs to be more clearly brought out in the paper as we have done in the introduction to the paper and in the section on review aims on page 4. It remains the case however that much community engagement with research proceeds as if the research is going to be done anyway and in a more instrumental fashion, and we feel it is important to understand the dynamics and impacts of this currently predominant practice.

1. **Understanding of the community engagement process** **:** **I found very limited discussion on what is community engagement. The process of community engagement is not discussed. I would have ideally preferred a little greater clarity on the *dynamic* nature of community engagement. As it presently reads, the construct of community engagement is under-theorized.**

 

 AUTHORS: On page 3 we attempt to summarise some of the range of activities that appear under the umbrella of community engagement, and we also highlight that there may be a smaller sub-set of relational mechanisms underpinning this diversity. In this sense we have indicated some fruitful lines of enquiry. However, these are often under-theorised in the present literature, which is one of our rationales for conducting the review. There are undoubtedly complex relational dynamics involved in engagement, some of which we have signalled in our visualisation in Figure 3. In addition, there is a prevailing ambiguity in the way engagement is presented, as to whether and how responsive the overall research process is to community influence and input. We expect to make contributions to better conceptualisation of engagement and understandings of the relational mechanisms underpinning it by conducting the review and to clarify how engagement works in practice.

  

1. **Towards deeper understanding of the multiple shades of community engagement** **:** **Given the formidable complexities that might underlie community engagement, I am sensitive to the challenges of understanding the CMO configurations underlying community engagement. I would recommend a more thoughtful discussion on why community engagement around research in malaria might be a little different than community engagement for other goals (for example, community engagement for gender empowerment). I found the discussion around the concept of community engagement quite mechanical.**

 

 AUTHORS: The review aims to focus on malaria as an initial pathfinder topic, both to draw on the considerable experience of the team and our content experts in this area, but also to make the review accomplishable, given the complexities noted by the reviewer. Indeed we expect there to be differences between engagement around malaria, and other diseases, such as HIV for example, as well as other initiatives with more of a social development focus. The role of stigma and power relations for example, may be less prominent within malaria research than within some other settings. The focus on engagement with health research, and the fact that engagement may have been typically deployed in a more instrumental way in this area, will need to be transparently flagged in the findings of the review. However, as noted above, we also aim in our search B to elucidate some broader engagement dynamics by drawing on literature from different disciplines and settings as far as is possible within the time and resources constraints of the review.

  

1. **A more explicit focus on LMIC contexts** **:** **Given that the research is being doing in LMIC settings, I did not find a discussion about the challenges of conducting research in LMIC setting. I found myself wondering which specific contexts in such settings will be explored. In what ways are the mechanisms associated with community engagement different in LMIC contexts?**

 

 AUTHORS: It is because we expect there to be particular characteristics of large health research programmes in LMICs that we chose to focus on such settings, in order to help clarify some of the contextual features that may have an important impact on engagement mechanisms, which may be quite different from those brought into play by research conducted in high income countries. In particular, the influence of colonial or neo-colonialism relations may be an important and distinctive part of LMIC settings, potentially demanding more comprehensive engagement processes. The LMIC focus is also partly about where the focus of the team’s ongoing work resides, In addition, it provides one way to focus the review to provide a manageable analysis process. Some relevant contextual features associated with LMIC settings that have been documented in the literature are highlighted in the background section on pages 3 and 4, but we await the review analysis to fully understand the influence of such factors and if and how different mechanisms may be implicated in LMIC settings.

  

1. **The need to more explicitly focus on values** **:** **Underlying much community engagement is a set of values. Community engagement can be quite exploitative or can be very respectful recognizing that the community is the seat of the decisions. The present version of the protocol takes a very instrumental approach to community engagement. I was somewhat surprised by an absence of any discussion around values in a realist synthesis protocol on community engagement.**

 

 AUTHORS: Much of the CE literature is aspirational, and it is not always clear at what level the values and principles being invoked actually affect practice. There may be an important role for making values explicit in community engagement, something which we hope the review will draw out if it is significant.

  

1. **Taking unintended outcomes seriously** **:** **There is a claim that the review will explore unintended outcomes, but there is very little discussion on how the authors plan to explore this. Most evaluations that might form the basis of such a synthesis do not often report on unintended outcomes; in this light it’s not clear how the authors intend to report on a synthesis of unintended outcomes.**

 

 AUTHORS: In the community engagement literature there is often a lack of clear statement of intended outcomes. As is typical in realist reviews, we will review the literature for examples of outcomes whether these are explicitly outlined at the outset or not, and seek to draw on relevant data explaining the contexts in which they have developed, and the mechanisms which may have been implicated. An example is provided by work documenting how the division between between participants and non-participants in vaccine trials in some settings has led to exacerbation of community differences and sometimes fuelled rumours about research (for eg. Gikonyo et al 2008/doi: 10.1016/j.socscimed.2008.02.003). In this way there may be interests that have been affected by research (usually negatively) that were not identified, acknowledged and safeguarded effectively through the community engagement. While unintended outcomes of community engagement, these processes are important to understand for the way they influence planned community engagement process and outcomes. The reviewer rightly points to the challenge that evaluation of community engagement is itself underdeveloped, and that finding unintended outcomes documented may be difficult. This issue of the availability of data on specific types of outcomes that we are interested in is an inherent limitation of literature reviews of any type – we can only analyse what is ‘out there’. As such, whilst we may make try our best to search for data on unintended outcomes, if we are limited by what is available, we will report this to be the case in future publications. However, at this stage of the review, scoping of the literature does indicate that there are data to draw on, and it remains important to consider outcomes beyond those explicitly aimed for in such a diverse and under-conceptualised field.

  

1. **The need for prioritization** **:** **Given the complexity of community engagement, wide varieties of CMO configurations might be possible. In the absence of clear prioritization, the complexity of having many CMO configurations with potentially limited evidence (across the multiple configurations of contexts and mechanisms) needs to be more explicitly discussed.**

 

 AUTHORS: We agree with the reviewer that there is a need for prioritisation. It is this that drove our initial focus on Malaria, in large health research programmes, in LMICs. At the same time, our concern that such a narrow focus might obscure the types of community outcomes such as empowerment, led us to our additional search components outlined above. We have attempted to strike a balance between making the review manageable and not neglecting engagement mechanisms that are outside of the mainstream. We also expect to have to progressively focus our review – as is expected of realist reviews [ http://ramesesproject.org/media/Realist_reviews_training_materials.pdf] . We will do so using a combination of the judgement of the project team’s content expertise, feedback and advice from our stakeholders and/or as directed by the data in the included documents.

  

1. **Interrogating the theory of change** **:** **Much of the theorizing is from a researcher’s perspective. The review presently does not have a strong plan to incorporate a community perspective to challenge, interrogate and refine the program theory. For example, I would have liked the authors to more clearly discuss how the community group members within the Wider Stakeholders Group can be utilized to refine the theory of change. **

 

 AUTHORS: Much of the published literature is from the researcher’s perspective. It is for this reason that we have a community consultation strand, which we expect to inform the programme theory and search strategy (including delving into different research paradigms) as the review progresses. We also aim to draw on the network of practitioners gathered through the MESH website and international community of practice. In addition, some of the published literature does include qualitative work that draws on interviews with a wider variety of research stakeholders, At the same time, there is value in greater clarity about what engagement from a researcher’s perspective actually aims at and actually achieves in practice in the dominant paradigm, in order to contextualise some of the volume of more aspirational claims made in the field.

**Comments on Rationale**

**While the rationale for the study is well described, it’s unclear in the absence of information on the outcomes how the proposed realist synthesis plans to achieve the objectives of the study. As pointed out earlier, the study also suffers from taking very much of a researcher’s perspective on potential benefits of research without sufficiently incorporating community members in either clarifying what community engagement means or in refining the theory of change.**

 AUTHORS: As noted above, given the diversity and complexity of the field, it is an important step to critically understand how engagement is being defined and developed in relation to some of the outcomes defined on page 3, in a major area such as engagement with large health research programmes in LMICs. Recognising the emphasis of existing literature on large research programmes and researcher’s perspectives led us to include a community consultation component which we expect to inform iterations of the programme theory, validation of emerging findings and review outputs. Recognising that there are always limits to what one review can cover, we believe the way we have designed the review will enable us to gain clarity on ‘common current practice’ around engagement in large health research initiatives, but that our search and review strategy should also enable us to draw out additional engagement and community dynamics that have been developed in other settings and can highlight alternatives approaches and related mechanisms.

**Appropriateness of Study**

**While there is much to be enthusiastic about the application of realist synthesis in understanding community engagement, many aspects of the proposed approach need to be further refined. The lack of a clear prioritization of CMO configurations, under-theorization of the dynamic nature of community engagement, and an absence of the discussion of the LMIC context that might be appropriate reduce my confidence in the proposed approach’s ability to refine and develop a useful program theory for community engagement. I would recommend focusing on some of these points.**

 AUTHORS: We appreciate the reviewers concerns. However, the very issues he has raised are in part related to the need to carry out a realist review – the need being that there is at present a poor understanding of the causal processes for many of the outcomes (of all types) of community engagement. It is potentially problematic to prioritise in advance CMO configurations as doing so may ‘shut down’ explorations of possibly more important causal explanations. We do intent to better theorise community engagement – albeit in the form of a realist programme theory – as the output of our review. We do intend to explore whether or not and what aspects of LMICs function as context within the CMO configurations we plan to develop. In addition, we feel that we have already addressed many other aspects of the issues raised in this comment in our responses above. These include questions raised around prioritisation and focus, around theorisation of community engagement and LMIC context. In this way we feel confident that the review can make an important contribution to developing the field.

**Details of the methods**

**As it presently reads, the details of the realist synthesis follow a general cookie-cutter approach and the complexity of conducting a realist synthesis for a complex area like community engagement especially in the domain of malaria research is not sufficiently considered. I also would argue that the Figure 3 as presented is not sufficiently clear as the starting point for developing an initial program theory**.

 AUTHORS: In some ways this is the nature of a protocol paper - we have to lay out a more generic process which we fully expect to need to be responsive and iterative as the review progresses depending on what emerges from the strands of literature and in response to stakeholder feedback. Figure 3, as noted in response to reviewer 1’s comments, is a collection of elements that are not yet configured and in this sense they do not represent a fully fledge programme theory. The elements gathered have sensitised us to particular issues to be attentive to during the analysis of the literature, including surfacing some potential mechanisms. We also wanted to include this diagram since the process of developing initial programme theory is often left unclear in realist review protocols, but we recognise we do need to be clearer about the exact status of the visualisation.

# Peer review: Reviewer response for version 1

**DOI:** 10.21956/wellcomeopenres.16697.r35686

**Published:** 2019-07-01

**Authors:** Maxine Whittaker

**Affiliations:** 1 Division of Tropical Health and Medicine, James Cook University, Townsville, Qld, Australia

In Goals of community engagement, the goal of using this method for co-design of health research has been left out. Increasingly there are researchers who are undertaking co-design with communities, the whole approach of participatory research.

 Outcomes could also include: community ownership, community empowerment, community leadership, sustainability. These are not considered. It may be that an intended or even unintended consequence of community engagement was a resultant community level outcome not just a malaria research or ethical obligation outcome.

 The dissemination activities should also be informed and co-designed with community groups. One example could be the recently formed Civil Society for Malaria Elimination. Dissemination, at least of briefs, in languages other than English would be advantageous for reach.

I confirm that I have read this submission and believe that I have an appropriate level of expertise to confirm that it is of an acceptable scientific standard.

# Response

**Published:** 2019-07-26

**Authors:** Bipin Adhikari

**Affiliations:** University of Oxford, UK

Dear Maxine,

 We are grateful to your suggestions and comments. Your suggestions were very helpful and we believe that these suggestions have improved our manuscript significantly.

 We have added our responses below with relevant corresponding changes in the manuscript.

 We look forward to your kind consideration.

 Sincerely yours,

 On behalf of co-authors,

 Bipin Adhikari

**In Goals of community engagement, the goal of using this method for co-design of health research has been left out. Increasingly there are researchers who are undertaking co-design with communities, the whole approach of participatory research.**

 AUTHORS: The reviewer usefully points out something that was discussed in our team but which we had not made explicit enough in the narrative. There are as far as we were aware very few examples of the co-design of large clinical trials and health research programmes. Many authors highlight this as an aspiration, and there are elements of research implementation that involve greater or lesser degrees of responsiveness to community concerns and ideas. Scoping for our programme theory suggested that there potentially were examples of outcomes of community empowerment linked to community engagement processes, but not usually associated with major programmes of health research, more often in the settings on international development or community development. It is for this reason that our search A looks at ‘common current practice’ of large clinical trials, while our search B aims to look beyond this to look at other paradigms and disciplines. We are aware that the causal logic and process and key related ‘mechanisms’ associated with community empowerment may not easily be found in the literature relating to engagement with clinical trials, and we may need to look elsewhere to find adequate specification of the relevant mechanisms and related data.

**Outcomes could also include: community ownership, community empowerment, community leadership, sustainability. These are not considered. It may be that an intended or even unintended consequence of community engagement was a resultant community level outcome not just a malaria research or ethical obligation outcome.**

 AUTHORS: Thanks for the comment that there may also be community level outcomes from engagement, some of which we have subsumed under our description of ethical outcomes (though these may not always be intended outcomes according to engagement plans and strategies). We have flagged these potential outcomes, in particular those of community empowerment and community capacity in our amended paragraph on p 3 referring to community engagement outside the settings of large health research programmes.

**The dissemination activities should also be informed and co-designed with community groups. One example could be the recently formed Civil Society for Malaria Elimination. Dissemination, at least of briefs, in languages other than English would be advantageous for reach.**

 AUTHORS: Thanks for the suggestions. We do have a community consultation component of the review, and this includes gathering input on dissemination and outputs. The addition of civil society Malaria forums as relevant stakeholders to develop outputs would be useful. The review process already aims to produce briefings and to support some networking to facilitate dissemination. We do not have the resources to do this in languages in addition to English in the project budget but there may be potential to develop some outputs in other languages depending on potential collaboration with other partners around the planned design of accessible review outputs.

# Peer review: Reviewer response for version 1

**DOI:** 10.21956/wellcomeopenres.16697.r35659

**Published:** 2019-06-25

**Authors:** Suzanne Day

**Affiliations:** 1 Institute for Global Health and Infectious Diseases, University of North Carolina at Chapel Hill, Chapel Hill, NC, USA

This study protocol describes the undertaking of a realist review of community engagement for malaria research in LMICs. The protocol begins by identifying the need for greater understanding of community engagement and how different contexts shape engagement outcomes and, subsequently, have implications for health research outcomes. It then describes the objectives of the review, and details the search strategies that will be used.

   

 Is the rationale for, and objectives of, the study clearly described?

- Partly. The rationale for the review is well articulated. Under “Review objective” on page 4, one of the stated goals is “To conduct a realist review to understand the ways in which community engagement contributes to intended and unintended outcomes” – but it is not clear whether the authors mean by "outcomes". Does this refer to outcomes for the health research for which community engagement is being conducted, or outcomes of engagement in general? It is not until page 8 that it is specified that both research outcomes and ‘ethical’ outcomes will be examined. Additionally, it is not clear what is meant by ‘ethical outcomes’ – does this refer to outcomes that enhance the ethical conduct of the research? More clarification would be helpful here regarding exactly how 'outcomes' are being defined in the context of this review. 

Is the study design appropriate for the research question?

- Partly. The authors are conducting a realist review, which is a method to develop/refine programme theory. However, it is unclear what ‘programme theory’ means in this context: is this a theory of how community engagement impacts health research? In other words, it is not clear what the programme theory will explain, so it is difficult to assess whether a realist review is the most useful study design. A scoping review would have similar elements (e.g. searching through both academic and grey literature; secondary searching of reference lists; consultation with stakeholders for additional inclusions, etc.) but without the goal of building theory, which may be more appropriate here if it is difficult to articulate exactly what the resultant theory is intended to explain. 

Are sufficient details of the methods provided to allow replication by others?

- Partly. There are some inconsistencies and missing information. For example, on page 5, the authors note that “The review will include all the pertinent documents published in English language only.” However, on page 7, they note that “The searches will not be limited by the date and (English) language, although they will be conducted in English.” Please clarify the search strategy and its language limitations. Additionally, location of community engagement should be part of the inclusion criteria for initial screening of literature in order to identify studies based in LMICs, which is a stated goal of the review. Please also specify the definition you are using to determine whether the literature is sourced from an LMIC context (e.g. World Bank definitions of LMICs?) 

Are the datasets clearly presented in a useable and accessible format?

- Not applicable. This protocol describes a literature review which is not yet completed, thus all data have yet to be collected. However, the final dataset (i.e. all literature included in the review) will be fully accessible as a part of the review article once it is completed. 

Additional Comments

- The conceptual distinction between “Outcomes” and “Contexts” is unclear in Figure 1. In the example given, it would seem that suspicion/openness to research is more an outcome than a context. Perhaps ‘Prior research experiences (e.g. historical mistrust due to past abuses/lack of engagement, vs. trust in research due to positive experiences)” would more accurately capture the concept of context here. In the smaller (definitional) wheel, you might specify that context refers to underlying social, political, historical, and economic factors within which engagement interventions are embedded.
- It is not always clear what the authors mean by “outcomes” – will the review focus on outcomes of engagement itself, or the outcomes (impact on) the health research for which engagement is conducted? For example, on page 4, 2 ^nd^ paragraph: The authors note “To date, however, there is a lack of analysis and explanation of how the factors of local social and cultural context have affected particular outcomes.” Which particular outcomes? Does this refer to research outcomes, or something else?
- Figure 3 is very difficult to understand due to a lack of definitions and clear labeling. For example, what do the blue bars signify? What is the difference between “conditions” and “contexts”? How are “interaction qualities” different from “contexts”?

I confirm that I have read this submission and believe that I have an appropriate level of expertise to confirm that it is of an acceptable scientific standard, however I have significant reservations, as outlined above.

# Response

**Published:** 2019-07-26

**Authors:** Bipin Adhikari

**Affiliations:** University of Oxford, UK

Dear Suzanne,

 We are grateful to your suggestions and comments. Your suggestions were very helpful and we believe that these suggestions have improved our manuscript significantly.

 We have added our responses below with relevant corresponding changes in the manuscript.

 We look forward to your kind consideration.

 Sincerely yours,

 On behalf of co-authors,

 Bipin Adhikari

 This study protocol describes the undertaking of a realist review of community engagement for malaria research in LMICs. The protocol begins by identifying the need for greater understanding of community engagement and how different contexts shape engagement outcomes and, subsequently, have implications for health research outcomes. It then describes the objectives of the review, and details the search strategies that will be used.

   

 Is the rationale for, and objectives of, the study clearly described?

- **Partly. The rationale for the review is well articulated. Under “Review objective” on page 4, one of the stated goals is “To conduct a realist review to understand the ways in which community engagement contributes to intended and unintended outcomes” – but it is not clear whether the authors mean by "outcomes". Does this refer to outcomes for the health research for which community engagement is being conducted, or outcomes of engagement in general? It is not until page 8 that it is specified that both research outcomes and ‘ethical’ outcomes will be examined. Additionally, it is not clear what is meant by ‘ethical outcomes’ – does this refer to outcomes that enhance the ethical conduct of the research? More clarification would be helpful here regarding exactly how 'outcomes' are being defined in the context of this review. **

 

 AUTHORS: Thanks for the comments. We discuss some of the main goals for engagement and their contributions to research related outcomes and ethical outcomes, and give some examples of the range of things articulated in the literature on page 3, (and it is the aim of the review to further sharpen the distinctions being made and review the evidence supporting the claims for engagements contribution to such outcomes). We have better signposted this account of the range of outcomes in the Review Objective sub-section on page 4 and with the amended heading on page 3.

**Is the study design appropriate for the research question?**

- **Partly. The authors are conducting a realist review, which is a method to develop/refine programme theory. However, it is unclear what ‘programme theory’ means in this context: is this a theory of how community engagement impacts health research? In other words, it is not clear what the programme theory will explain, so it is difficult to assess whether a realist review is the most useful study design. A scoping review would have similar elements (e.g. searching through both academic and grey literature; secondary searching of reference lists; consultation with stakeholders for additional inclusions, etc.) but without the goal of building theory, which may be more appropriate here if it is difficult to articulate exactly what the resultant theory is intended to explain. **

 

 AUTHORS: Thanks for the comments. We have added a definition of what a programme theory is as understood within realist reviews on page 11 of the revised manuscript. Despite the variability of the literature there are also some fairly well-developed claims linked to instrumental outcomes for improvement of research, and for ethical outcomes, including some of those we highlight in our introduction (and as we also we note in our introduction, these outcomes are sometimes conflated). There is also some considerable empirical work in these areas, even while it remains relatively disparate. It is precisely the causal explanations of how engagement process work that we are interested in clarifying within our programme theory, as a way to begin to move the field forward and to underpin more effective evaluation, and this is what makes a realist review the synthesis framework of choice

**Are sufficient details of the methods provided to allow replication by others?**

- **Partly. There are some inconsistencies and missing information. For example, on page 5, the authors note that “The review will include all the pertinent documents published in English language only.” However, on page 7, they note that “The searches will not be limited by the date and (English) language, although they will be conducted in English.” Please clarify the search strategy and its language limitations. Additionally, location of community engagement should be part of the inclusion criteria for initial screening of literature in order to identify studies based in LMICs, which is a stated goal of the review. Please also specify the definition you are using to determine whether the literature is sourced from an LMIC context (e.g. World Bank definitions of LMICs?) **

 

 AUTHORS: Thanks for pointing out some of these inconsistencies that we need to address. The searches will be limited to the English language. We will restrict the initial searches to LMIC countries and use definition of LMICs as outlined by the World Bank and have added this detail into the paper on page 7. However, where we lack sufficient data to contribute to testing and refinement of particular aspects of the programme theory we may seek to draw on work outside LMIC contexts where it can provide a contribution to development of programme theory, as is common practice in realist reviews

**Are the datasets clearly presented in a useable and accessible format?**

- **Not applicable. This protocol describes a literature review which is not yet completed, thus all data have yet to be collected. However, the final dataset (i.e. all literature included in the review) will be fully accessible as a part of the review article once it is completed. **

**Additional Comments**

- **The conceptual distinction between “Outcomes” and “Contexts” is unclear in Figure 1. In the example given, it would seem that suspicion/openness to research is more an outcome than a context. Perhaps ‘Prior research experiences (e.g. historical mistrust due to past abuses/lack of engagement, vs. trust in research due to positive experiences)” would more accurately capture the concept of context here. In the smaller (definitional) wheel, you might specify that context refers to underlying social, political, historical, and economic factors within which engagement interventions are embedded.**

 

 AUTHORS: We have provided a glossary to explain what is meant by context and outcomes in a realist review (under methods section). It is also worth noting that this manuscript is a protocol and we have not completed the review yet. At this early stage in the review, we have provided Figure 1 only as an illustrative and speculative example of mechanisms ( http://ramesesproject.org/media/Realist_reviews_training_materials.pdf). In our simplified diagram we wanted to show that the prevailing context of either openness to research or suspicion to research would affect whether the mechanism of willingness to place trust comes into play (these may indeed be due to prior research experiences or historical mistrust, but it is the prevailing context we are signalling here). And in the smaller wheel, we wanted to highlight that what are considered contexts in realist analysis is the sub-set of the setting (often called wider context) that actively influences the mechanism of interest. Social, political economic and historic factors are present in the setting, but the notion of context aims to flag those aspects that are particular salient for the mechanism of interest (see related comment below about Figure 3)

- **It is not always clear what the authors mean by “outcomes” – will the review focus on outcomes of engagement itself, or the outcomes (impact on) the health research for which engagement is conducted? For example, on page 4, 2 ^nd^paragraph: The authors note “To date, however, there is a lack of analysis and explanation of how the factors of local social and cultural context have affected particular outcomes.” Which particular outcomes? Does this refer to research outcomes, or something else?**

 

 AUTHORS: As noted in response to previous comments, we gave an overall treatment of the diversity of outcomes on page three. In addition, different studies give accounts of contributions to different outcomes and part of the aim of the review is to organise some of these distinctions and gather evidence for the mechanisms that underpin some of them. This is part of the inconsistency that the review is attempting to address. We have flagged this issue of diversity of outcomes at the outset of the paper on page 3, while also identifying some of the documented range. In addition as noted above, we flag this again when mentioning outcomes in the ‘Review Objective’ sub-section on p 4 to remind the reader of this diversity.

- **Figure 3 is very difficult to understand due to a lack of definitions and clear labeling. For example, what do the blue bars signify? What is the difference between “conditions” and “contexts”? How are “interaction qualities” different from “contexts”?**

 

 AUTHORS: Realist reviews are routinely expected to produce an initial programme theory. Such a theory is not expected to be definitive but to provide an illustrative overview of the possible issues and ideas that may need to be considered with the review. As such, any initial programme theory does often lack the precision and clarity of the finalised programme theory expected at the end of a realist review (Reference: http://ramesesproject.org/media/Realist_reviews_training_materials.pdf). We wanted to be as transparent as possible and include the visualisation since this was part of the initial process of identifying programme theory and this is often not made visible in protocol papers. However, the visualisation is more a collection of elements to help us focus searches and analysis, rather than a more strictly defined diagram as is highlighted in the paper. Given the tendency for it to be interpreted as the latter, this does need more emphasis and so we have attempted to do this on p6. In addition (as noted above in response to the previous comments about Figure 1) in Figure 3 our programme theory highlights some things as background ‘conditions’ while others are flagged as likely contextual influences. We have added some clarification on this distinction on page 6

# Peer review: Reviewer response for version 1

**DOI:** 10.21956/wellcomeopenres.16697.r35669

**Published:** 2019-06-25

**Authors:** Rosemary Musesengwa

**Affiliations:** 1 Department of Psychiatry, University of Oxford, Oxford, UK

This is a pertinent review in the field of Community Engagement (CE). Employing a realist perspective is a novel and appropriate method for CE. Their review of the literature is well structured and the need for a realist perspective is well justified. The proposed review shows considerable engagement with the current literature drawing robust conclusions. I agree that current literature on CE does not present a coherent evidence base that will enhance understanding of how different CE mechanisms contribute to research outcomes.

 It is a very welcome review at a time when it seems now CE has become rather a technical imperative rather than an ethical imperative as it rightly be. I believe the lack of coherent literature has led to researchers relying on random literature which might not lead to desired or intended outcomes.

 The methodology proposed is logical and appropriate. The authors have a clear picture of what the review will entail. I do have reservations on the selection of the broader stakeholders from snowballing. Snowballing for stakeholders will ultimately lead to people who have the same mindset and view of an issue and can be extremely biased at most. A more structured/stratified approach is recommended drawing from all schools of thought from within which snowballing may be employed.

 Limitations of the review are well articulated by the authors. The difficulty of searching for CE literature as most of the literature is rarely written with the intention of illustrating CE activities/mechanisms but are usually implied in other types of manuscripts, should be underscored.

 The review promises to bring clarity and understanding to CE literature.

I confirm that I have read this submission and believe that I have an appropriate level of expertise to confirm that it is of an acceptable scientific standard.

# Response

**Published:** 2019-07-26

**Authors:** Bipin Adhikari

**Affiliations:** University of Oxford, UK

Dear Rosemary,

 We are grateful to your suggestions and comments. Your suggestions were very helpful and we believe that these suggestions have improved our manuscript significantly.

 We have added our responses below with relevant corresponding changes in the manuscript.

 We look forward to your kind consideration.

 Sincerely yours,

 On behalf of co-authors,

 Bipin Adhikari

**This is a pertinent review in the field of Community Engagement (CE). Employing a realist perspective is a novel and appropriate method for CE. Their review of the literature is well structured and the need for a realist perspective is well justified. The proposed review shows considerable engagement with the current literature drawing robust conclusions. I agree that current literature on CE does not present a coherent evidence base that will enhance understanding of how different CE mechanisms contribute to research outcomes.**

****

**It is a very welcome review at a time when it seems now CE has become rather a technical imperative rather than an ethical imperative as it rightly be. I believe the lack of coherent literature has led to researchers relying on random literature which might not lead to desired or intended outcomes.**

 AUTHORS: Thank you for the comments.

**The methodology proposed is logical and appropriate. The authors have a clear picture of what the review will entail. I do have reservations on the selection of the broader stakeholders from snowballing. Snowballing for stakeholders will ultimately lead to people who have the same mindset and view of an issue and can be extremely biased at most. A more structured/stratified approach is recommended drawing from all schools of thought from within which snowballing may be employed.**

 AUTHOR: Thank you for the useful suggestion. In addition to an element of snowballing we are aiming to find stakeholders willing to give input and review interim outputs and iterations of the programme theory from a number of different disciplines (public health, epidemiology, biomedicine, psychology, social science, anthropology, critical community practice….) and a range of different positionings in relation to engagement as a practice (funders, implementers, research managers, engagement practitioners and community representatives). We have amended the paper on page 6 to highlight that we will pro-actively seek to find inputs from this profile of stakeholders beyond our existing networks of contacts, through current literature in different fields.

**Limitations of the review are well articulated by the authors. The difficulty of searching for CE literature as most of the literature is rarely written with the intention of illustrating CE activities/mechanisms but are usually implied in other types of manuscripts, should be underscored.**

****

**The review promises to bring clarity and understanding to CE literature.**

 AUTHORS: Thanks
